# Reconstruction of a Damaged Lower Polar Artery for Kidney Transplantation Using Tubularised Donor Aorta

**DOI:** 10.1155/2017/3532473

**Published:** 2017-10-16

**Authors:** A. J. Vicéns-Morton, C. Callaghan, J. Olsburgh

**Affiliations:** Guy's and St Thomas NHS Foundation Trust, Renal Department, London, UK

## Abstract

**Introduction:**

Live donors, extended donor criteria, and the maximum usage of organs with anatomical variants are some of the mechanisms used to increase the number of organs available.

**Case:**

We present the case of a kidney transplant, in which the organ had an iatrogenic injury to a lower pole arterial branch during retrieval. The donor was a 35-year-old male (DCD, Maastricht III). The right kidney was accepted; it had three veins in a single cava patch and three renal arteries, the main artery with aorta patch that is 8 cm long. A small lower pole artery was sectioned during retrieval surgery at approximately 1 cm from its origin as well as a third small mid-lower pole artery. The lower pole damaged artery was reconstructed using tubularised aorta patch to a total length of 5 cm. No additional donor vessels had been sent. After construction of the tubulised aorta, E-E anastomosis to the damaged polar artery was done with interrupted 7-0 Prolene sutures.

**Conclusion:**

While the waiting list for a kidney continues to rise and we continue to have organ shortness, vascular retrieval injury should not be an absolute contraindication for transplant.

## 1. Introduction

Kidney transplantation is the gold standard renal replacement therapy for patients suffering from end-stage renal disease (ESRD); it is more cost effective than haemodialysis and provides a better quality of life [1]. With the advance of medicine and the increased life expectancy, the demand for organs has increased globally.

Many novel techniques and protocols have been introduced in order to increase the number of organs available. Live donors were responsible for 31,5% of kidney transplants in the UK last year (according to NHS-BT, 2972 kidneys were transplanted; 937 were from a live donor). Also, the use of “extended donor criteria” and the maximum usage of organs with anatomical and/or vascular variants are mechanisms used to increase the number of organs available, although this makes great demands on the transplant surgeons.

We present the case of a kidney transplant, in which the organ had an iatrogenic injury to a lower pole arterial branch occurring during retrieval. Reconstruction was performed using donor's tubularised aortic patch and the recipient has had a satisfactory outcome. To the best of our knowledge, this is the first case report of this technique.

## 2. Case

The donor was a 35-year-old male, Maastricht category III, who died after cardiac arrest (DCD) due to intracranial haemorrhage secondary to trauma. The right kidney was sent to another centre first and declined due to vascular damage. It then went through the fast-track scheme and was accepted by our team at Guy's Hospital. Once we had the kidney, the initial evaluation showed a right kidney, with three veins in a single cava patch, three renal arteries, the main artery with aorta patch that is 8 cm long, a small lower pole artery, which was sectioned during retrieval surgery at approximately 1 cm from its origin, and a third small mid-lower pole artery. The ureter had bifid renal pelvis.

During bench surgery the kidney was well perfused well with Soltran solution. From the three veins in the single cava patch, it was decided to ligate the posterior branch in order to allow the single patch to be more mobile. The small mid-lower pole artery was already damaged and was deemed unreconstructable and was therefore tied off. The main artery was left with a 1 cm aortic patch. The lower pole damaged artery was reconstructed using tubularised aorta patch to a total length of 5 cm. No additional donor vessels had been sent.

Although the inferior epigastric artery is sometimes preferred for this kind of reconstruction, in this case, it would probably have been too small for the reconstruction; also, the availability of a long and healthy aortic patch and the fact that during benching we did not know the status of the recipient vessels made us decide to take this approach.

**Figure 1:**
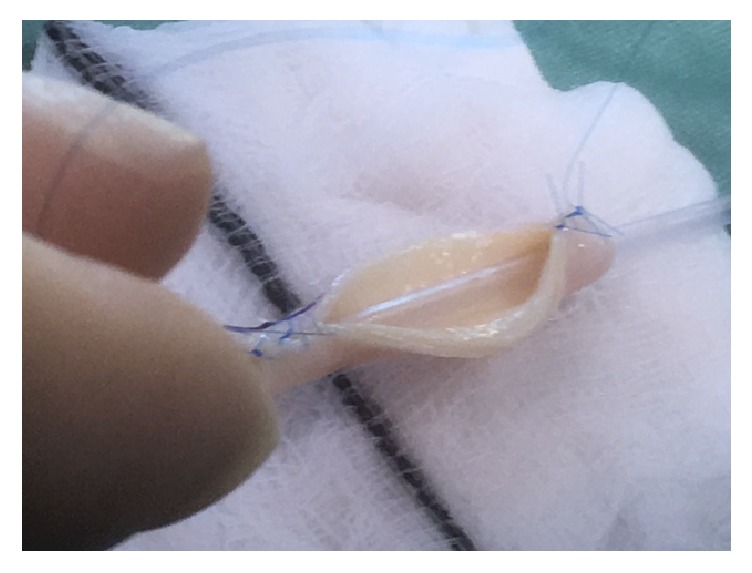
Aortic patch over Nelaton 8 ch catheter.

**Figure 2:**
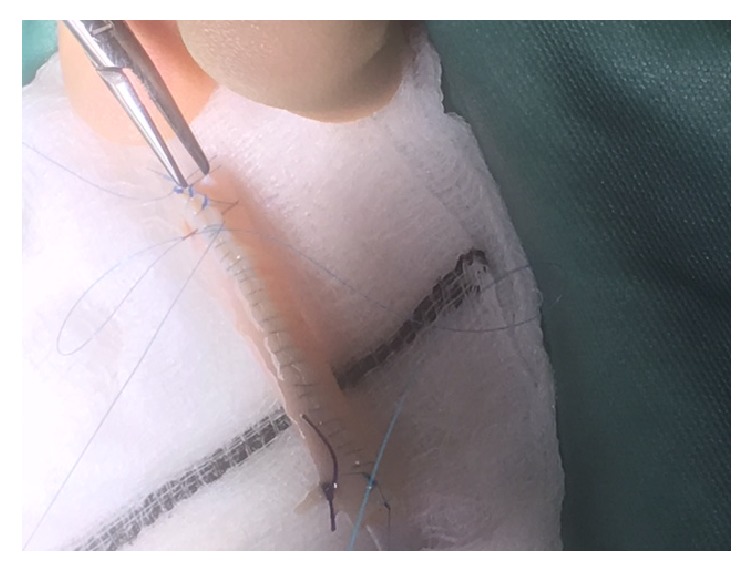
Aortic patch over Nelaton 8 ch catheter.

**Figure 3:**
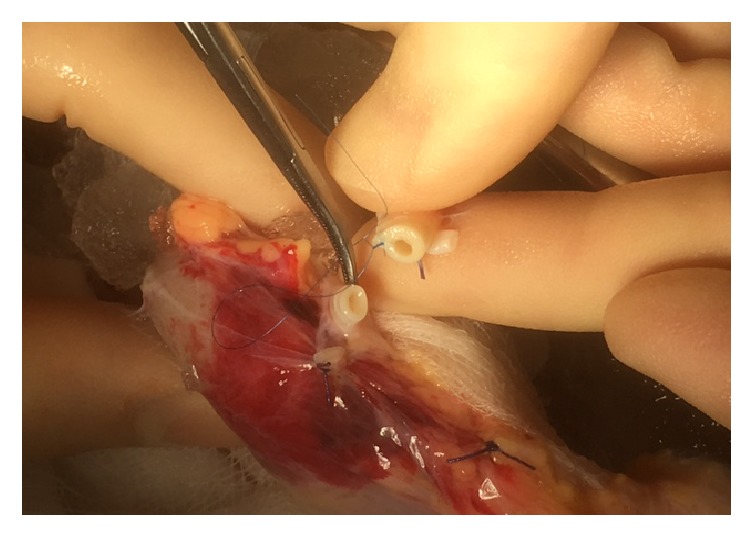
Start of the E-E anastomosis between tubulised aortic patch and polar renal artery.

**Figure 4:**
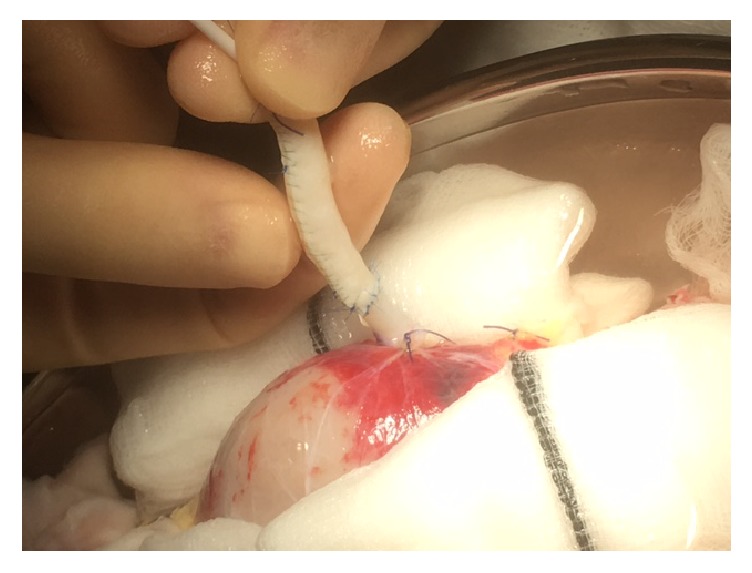
Completed E-E anastomosis between tubulised aortic patch and polar renal artery.

The reconstruction was carried out with an aorta patch that is 5 cm in length that was part of the main artery patch of the organ. Using an 8 ch Nelaton bladder catheter as a mold a 5 cm long aorta segment was tubularised using 3 7-0 Prolene interrupted stiches in the distal area to avoid stenosis and Prolene 7-0 continuous suture in the rest of the patch to minimize bleeding risk. After construction of the tubulised aorta, E-E anastomosis to the damaged polar artery was done with interrupted 7-0 Prolene (Figures 1–4 showing steps of the vascular reconstruction).

The recipient was 68-year-old male, with past medical history of ESRD secondary to IgA nephropathy on peritoneal dialysis and no other medical issues nor surgical procedures. After a detailed discussion with the patient, regarding the benefits and the risks due to the surgery and donors/organ characteristics, the patient was happy to proceed and signed the consent.

**Figure 5:**
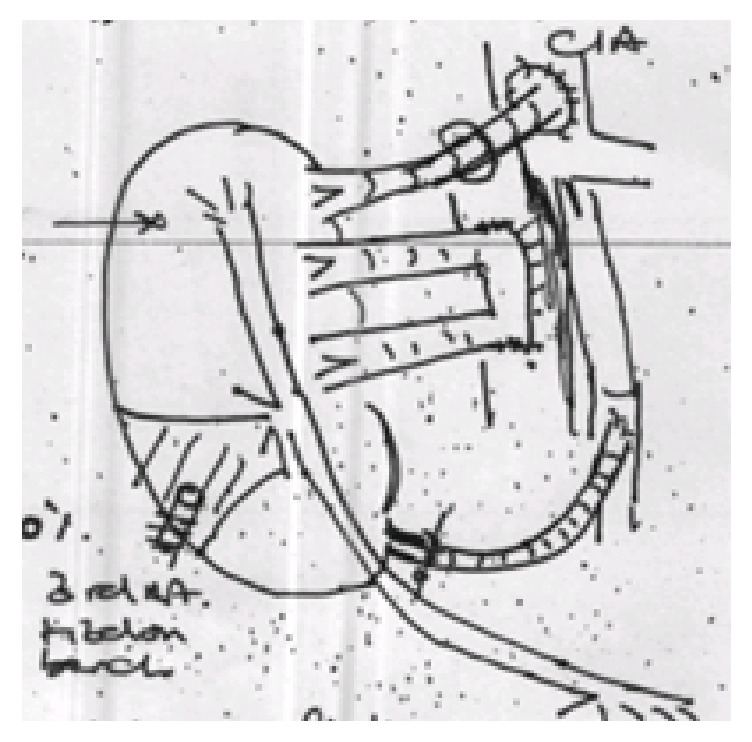
Drawing of the transplant showing ischemic area and all three vascular anastomoses.

**Figure 6:**
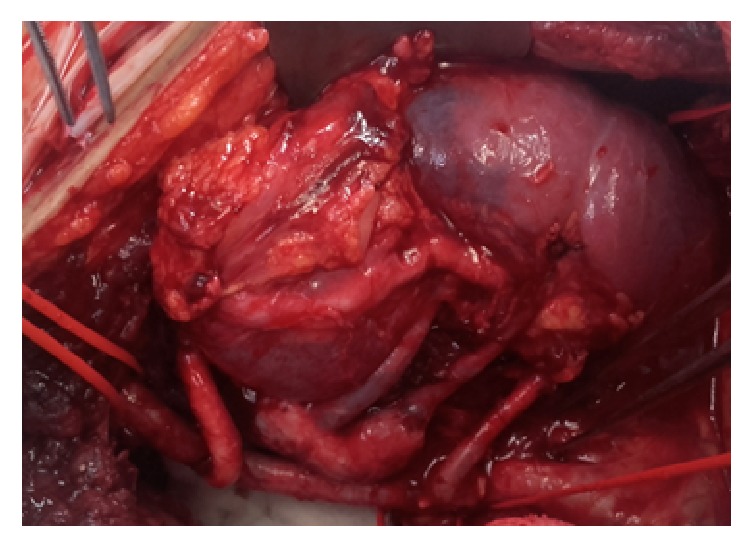
Kidney after reperfusion, all three vascular anastomoses.

**Figure 7:**
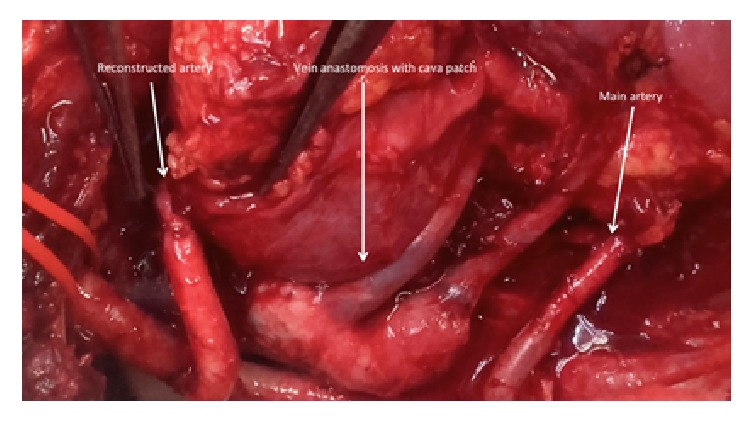
Close look at all three vascular anastomoses after reperfusion.

Transplant surgery was performed in the right iliac fossa with an extraperitoneal approach to the iliac vessels. The common cava patch was anastomosed to the recipient's external iliac vein. The main artery was anastomosed to the common iliac artery. Finally the reconstructed artery with the tubulised patch anastomosed the external iliac artery. After completion of all three vascular anastomoses the kidney was reperfused (Figures 5, 6, and 7). The ureter was anastomosed to the bladder over a double J stent. A Robinson drain was left and wound was closed. The cold ischemic time was 27 hours and 15 minutes and warm ischemic time was 75 min.

There were no immediate postoperative complications. The first ultrasound was performed two hours after finishing the surgery in the recovery ward, showing good perfusion of the kidney.

After 48 hours a second ultrasound was performed. The presence of a superficial haematoma within the subcutaneous tissues and slightly reduced perfusion within the interpolar region was reported; otherwise appearance of the transplant kidney was satisfactory.

Haemoglobin fell from 11.2 (preoperatively) to 6.4 gr/dl. A decision to transfuse two units of blood cells and a relook surgery was reached. There was subcutaneous and perigraft haematoma and no active bleeding. The three vascular anastomoses were identified, both arteries, main and reconstructed, had good thrill, and the vein was soft and had good outflow. A new drain was left at the surgical site and wound closure was performed.

Three days later, the ultrasound was repeated showing that the right iliac fossa transplant kidney had normal cortical thickness and appearance. There was no pelvicalyceal dilatation. The previously demonstrated superficial collection was no longer present. There was satisfactory global vascularity. The sampled interlobular and arcuate vessels demonstrate normal flow with resistive indices between 0.65 and 0.8. The two renal arteries and veins had normal spectral waveforms, with a final impression of normal appearances of right iliac fossa transplant kidney.

Ureteric stent was removed at week 4 after transplant. Three months after transplantation the patient was stable and had no dialysis requirements with creatinine of 187.

## 3. Discussion

Donor kidneys with damaged vessels are often declined when offered for transplantation because of increased risk of thrombosis, bleeding, or compromised renal function. The use of these organs represents a technical challenge to the transplant surgeons [2].

The retrieval technique has a definitive impact on the final outcome after transplantation. During harvesting of the kidneys all the structures should be carefully identified. Renal arteries should be preserved and explanted with a common aortic patch when possible. Polar arteries may originate far away from the main renal artery, for example, common iliac artery. If a common patch is impossible to preserve, patches for each artery should be obtained. All the arteries and veins as well as existing patches must be dissected from perivascular tissue. Cannulation of every single artery and perfusion should be performed ex vivo immediately [1].

Some of the problems that can be found in the organs are arteriosclerosis, renal artery stenosis, and intimal desquamation requiring shortening of the renal artery. Generally patches with severe arteriosclerosis and intimal lesions should be removed [1].

The presence of vascular anomalies, disease of the vessels, or iatrogenic injury to the vasculature during organ procurement may necessitate reconstruction of these vessels ex vivo prior to transplantation to salvage these allografts for transplant [3].

Some of the techniques most frequently used for reconstructing vessels include end-to-side (E-S) anastomosis of a branch vessel to the main artery, anastomosis of a branch vessel to the aortic patch, side-to-side (S-S) anastomosis of two aortic patches, anastomosing two renal arteries together to form a single stem, end-to-end (E-E) anastomosis of the main renal artery to a donor arterial graft, E-E anastomosis of the main renal artery to a synthetic graft, and anastomosis of a polar artery to the inferior epigastric artery of the recipient [3].

Reconstruction of the donor renal artery is associated with higher rates of arterial complications postoperatively [3]. Ex vivo reconstruction of the artery is a significant risk factor for the development of arterial thrombosis and arterial stenosis postoperatively [4]. The renal artery stenosis, the most frequent vascular complication after renal transplant and vascular reconstruction, increases this risk [3].

The 5-year graft survival for kidneys with reconstructed renal arteries was 84.3% in deceased donor renal transplantations (86.1% without arterial reconstruction) [5].

The gold standard for evaluation of the perfusion after renal transplantation is colour duplex sonography. The method is cost effective and suitable to detect vascular problems [6]. Further imaging diagnostic tools include angiography and renal scintigraphy. When doubts persist, a second surgical look should be indicated [1].

## 4. Conclusion

While the waiting list for a kidney continues to rise and we continue to have organ shortness, vascular retrieval injury should not be an absolute contraindication for transplantation. There are many different options and techniques available in order to repair vascular damage. We describe a new technique using the aortic patch. Which technique to use will depend on the vascular defect we are dealing with and the availability of extra vessels sent by the retrieval team.
